# Six-Step Model of Nature-Based Therapy Process

**DOI:** 10.3390/ijerph17030685

**Published:** 2020-01-21

**Authors:** Kyung Hee Oh, Won Sop Shin, Tae Gyu Khil, Dong Jun Kim

**Affiliations:** 1 Department of Forest Therapy, Chungbuk National University, Cheongju, Chungbuk 28644, Korea; okh7864@naver.com (K.H.O.); shinwon@chungbuk.ac.kr (W.S.S.); ktg0704@hanmail.net (T.G.K.); 2 Department of Forest Science, Chungbuk National University, Cheongju, Chungbuk 28644, Korea

**Keywords:** natural environment, forest therapy, theoretical model, stress, public health

## Abstract

Several studies have confirmed that the natural environment has psychophysiological healing effects. However, few studies have investigated the healing process involved in the effect of the natural environment. To date, no theoretical model on the nature-based therapy process has been clearly established. Thus, the aim of this study was to develop a theoretical model of the nature-based therapy process by analyzing individual empirical data. Research materials were 180 self-reported essays on “Forest Therapy Experiences” submitted to the Korea Forest Service. This study was conducted based on grounded theory. Data were analyzed through open coding. A total of 82 concepts, 21 subcategories, and six categories were derived. Results revealed that the nature-based therapy process contained six categories: Stimulation, acceptance, purification, insight, recharging, and change. When in the natural environment, participants first experienced positive emotional change, followed by cognitive and behavioral changes. Based on these results, a nature-based therapy process model was derived. This study revealed that the nature-based therapy process did not consist of just a single element or step, but involved an integrated way of healing with emotional and cognitive changes. This study is significant in that it derives a theoretical model of the nature-based therapy process with comprehensive mechanisms. Further research is needed to establish more systematic theoretical model.

## 1. Introduction

Modern people are exposed to various problems and diseases due to rapid social and lifestyle changes. In particular, stress is a major threat to the quality of life and health. It is strongly associated with psychological and physical diseases [1]. Stress is caused by the stimulus of any object or situation we encounter [2].

When the body is exposed to external stimuli, the brain recognizes them as threats and activates the autonomic nervous system inducing a “flight-or-fight” response [3]. Stress can be divided into distress and eustress [4]. Moderate stress in daily life plays a positive role by providing energy, stimulation, and motivation in life. Hormones associated with stress can protect the body in the short-run and promote adaptation. However, if stress hormones are built up in the body without being released, they can lead to chronic stress which might destroy the immune system and cause various ailments [3]. Stress that continues for a prolonged period has negative effects on health [3,5].

Responses to stress and coping strategies vary from person to person [6]. Failure to cope with negative and chronic stress can lead to mental and physical illnesses [7] including mental disorders (such as depression) and physical disorders (such as angina).

Relieving stress has emerged as an important issue to improve health and quality of life. Interest in nature-based therapy is increasing because having contact with nature can relieve stress with a positive psychophysiological effect. Many studies have reported the positive effect of natural environments. There are several major theories related to nature-based therapy. Ulrich’s psycho-evolution theory (PET) [8] and Kaplan & Kaplan’s attention restoration theory (ART) [9] are two core theoretical models on nature’s ability to relieve stress in humans. Ulrich et al. [8] have suggested that humans have evolved psychologically and physiologically so that they can function well in the natural environment. Thus, humans can relieve their stress much faster when they are in nature rather than in cities. According to the ART [9], the natural environment can rapidly recover human attention by restoring and renewing the capacity depleted by fatigue and stress. Continued attention and fatigue can degrade our ability to solve problems and cause various negative emotions. Kaplan & Kaplan [9] have suggested that the natural environment can resolve health problems of modern people’s daily lives caused by stress and fatigue.

These two theories are related to Wilson’s ‘biophilia’ hypothesis [10]. ‘Biophilia’ means ‘love for life’. The hypothesis assumes that humans have attachments to nature with a nature-returning instinct because humans have lived in nature for a long time. In other words, since the physiological function of human beings is suited to nature, they can instinctively feel relaxed and bonded when they have contact with nature. Thus, stress may be reduced, leading to positive psycho-physiological responses. The ‘topophilia’ hypothesis expands the idea beyond the ‘biophilia’ hypothesis [11]. ‘Topophilia’ means ‘love for places’ formed by experiences. This means that humans can form an affiliation with the natural environment through acquired learning. This hypothesis explains human interests and positive feelings about nonliving components (such as water and stones) and living elements in the biophilia hypothesis.

Many studies support these nature recovery theories in various ways. Ulrich [12] compared the number of hospital stays, patient records, and analgesic doses from 1972 to 1982 at a hospital in Pennsylvania for patients undergoing operations. He assigned patients to two different groups: A group with window view rooms where patients could see trees and another group with windows facing a brick wall. Results showed that the group with a tree view through the window had fewer days of hospitalization and took less analgesic than the group with a brick wall view, indicating that a view of the natural environment could influence the postoperative recovery of patients.

Hartig et al. [13] conducted a comparative study on stress levels and attention recovery with 112 adults in natural and urban surroundings by repeatedly measuring subjects’ blood pressure and emotional factors to test how human restoration values are associated with nature and urban environments. They found that walking in a natural environment could reduce negative emotions, increase positive emotions, and improve work performance. Shin [14] has examined how the forest around the workplace affects job satisfaction and stress of workers. Results showed that workers with a forest around their workplace had higher job satisfaction with lower levels of stress and lower turnover rates than those without forest surroundings.

Validation studies about the physiological effect of forest baths have been actively conducted in Japan. Park et al. [15] assigned 280 subjects in their twenties to 12 groups and had them walk around forests and urban areas. Their cortisol levels, blood pressures, pulses, and heart rates were then measured. Their results showed that forests lowered cortisol concentration, pulse rate, and blood pressure, suppressed sympathetic nerves, and activated parasympathetic nerves compared to urban surroundings. Li [16] has investigated the effects of forest bathing on human immune function. After male and female subjects traveled in the forest for two nights and three days, their blood and urine samples were collected on the second and third day during the trip and at seven and 30 days after the trip. Results showed that the number of NK (Natural Killer) cells was significantly higher than usual while cortisol concentrations were lower than those from their usual daily life for both men and women. The increase in NK cells lasted up to 30 days after the trip, confirming that forest bathing could lead to the maintenance of high levels of NK cell activity. Morita et al. [17] measured and compared the effects of forest baths on 498 participants. They reported decreases in hostility and depression with increases in vitality when they visited the forest, suggesting that forests might be particularly effective in reducing chronic stress and acute emotions and that forest baths could help reduce the risk of diseases associated with psychosocial stress.

Studies on stress and cognitive recovery in natural environments have also been performed in various ways. Tryvainen et al. [18] surveyed 77 participants about their recovery after visiting urban buildings, urban parks, and urban forests in Helsinki. They reported that visiting urban forests had a positive effect on stress relief and cognitive recovery as compared to visiting a man-made environment. Hansman [19] assessed the recovery effect of urban forests and urban parks in Zurich, Switzerland. The results revealed that suffering from headaches and stress decreased significantly while feelings of being well-balanced increased significantly. The recovery ratio for stress was 87% and the reduction ratio for headaches was 52%.

These findings confirmed that a green environment could promote health and recovery in humans. In addition, activities in a natural environment can significantly improve self-esteem and reduce overall negative feelings [20]. Kuo & Sullivan [21] have reported that people living in desolate buildings of an inner city have higher mental fatigue, aggression, and violence than residents living in rural areas. Their results indicated that having contact with nature could reduce mental fatigue and mitigate aggression and violence. Kuo [22] has found that residents without a natural environment tend to be lazier and regard problems more seriously than residents with a natural environment. In addition, the natural environment can reduce mental fatigue in residents, improve their efficiency, and enhance their problem-solving abilities in everyday life [22].

It has also been reported that green environments have positive effects on cognitive abilities of children. Wells [23] investigated the relationship between the natural environment and the cognitive functions of children from families with low-incomes in urban areas and reported that cognitive function can be improved to a high level after children move to a house with a green environment.

In addition, forests can affect spiritual aspects and enhance well-being [24]. Previous studies have also reported that forests can provide an experience of transcendent moments [25]. Mackerron & Mourato [26] have used GPS to study the relationship between subjective well-being and personal environment and found that participants are happier in a green or natural living environment than in an urban environment, further emphasizing the relationship between the natural environment and happiness.

Previous studies [13,14,15,16,17,18,19,20,21,22,23,24,25,26] have shown that having contact with nature is psychologically and physiologically effective in relieving stress, reducing depression and negative emotions, and increasing positive emotions. Nature also has a cognitive recovery effect. It can improve concentration and daily problem-solving abilities. Recovery in nature is cumulative. The more it is repeated and the greater the number of visits to a green space, the less likely the incidence of stress-related diseases [27]. The higher the ratio of greenery and access to greenery, the greater the physical health and mental well-being [28,29,30,31] and the less the health disparity caused by income differences [32].

These results suggest that the natural environment has positive effects on public health, indicating that nature-based therapy can be utilized as a strategy to prevent diseases. Despite these positive effects of the natural environment, it is currently unclear why and how recovery occurs in nature. Previous studies were mostly effect-oriented. Only a few studies have determined how physiological, mental, and social changes proceed in natural environments. To date, there is no theoretical model to explain the nature-based therapy process. For nature-based therapy to be recognized and further developed for use in the field of public health, it is necessary to develop theoretical models of nature-based therapy processes and mechanisms. Thus, the aim of this study was to develop a theoretical model of the nature-based therapy process by analyzing empirical data.

## 2. Materials and Methods

### 2.1. Data Collection

Data collected for this study were 180 self-reported essays entitled “forest therapy experiences” that were submitted to the Korea Forest Service from 2014 to 2015. The Korea Forest Service has hosted an annual public essay contest on forest therapy experiences in order to promote and draw public attention to the importance of forests and natural recovery effects. Topics of essays used in this study were all related to forest therapy, such as overcoming diseases (cancer, depression, etc.), recovering from various addictions, and resolving family problems. Those without an experience of natural recovery effects were excluded. The text format was free prose which was about 3–4 A4 sheets in length. The Korea Forest Service stated that “all submitted essays will not be returned and may be used for public interest.” Among these works submitted, excellent ones were selected and published in a book. Data used for this study were not raw data. They were edited and compiled. The 180 essays collected were reconstructed with all personal information (national resident numbers, names, phone numbers, etc.) removed and assigned serial numbers. Therefore, data of this study did not include any information disclosing personal identity. Essays cited were taken from those already published in a book from 2014 to 2015. This study did not directly observe or interview individuals. Their essays used in this research were open to the public. All personal information was removed during the editing process. In principle, the use of materials that are open to the public does not require Institutional Review Board (IRB) review. Therefore, this study was not reviewed by the Institutional Review Board (IRB).

### 2.2. Composition and Contents of Study Data

Study data were composed of stories of experience of individuals who visited the natural environment on their own and experienced a recovery. Participants described their own natural recovery experience in a free format. Data included information about what participants felt and how they were moved when they first visited the natural environment, where they visited, main symptoms, causes of stress, changes in body and mind, and the healing process. In addition, these essays specifically described motives for changing one’s perspectives and thoughts while subjects were in the natural environment, the state of psychological change, and changes in life and values after the recovery. This study identified some patterns and common phenomena in the healing process. These patterns and phenomena were then developed into concepts that could infer the nature-based therapy process.

### 2.3. Methods

Because nature-based therapy proceeds through a very complex mechanism, existing quantitative research methodologies alone are limited in developing a theory encompassing multiple aspects of the nature-based therapy process. To develop a theory on the nature-based therapy process, there should be sufficient investigations on nature-based therapy phenomena. Therefore, qualitative studies are needed to complement quantitative studies. The research method was based on the grounded theory of Strauss & Corbin [33], a qualitative methodology. Grounded theory is a systematic way of developing a theory from empirical data [33]. The analysis was carried out in four stages of open coding with one forest therapy consultant and one qualitative researcher to eliminate possible biases from researchers and increase the objectivity (Table 1).

The sequence of analysis was as follows. First, the overall feeling and perceptions of healing experiences were derived by repeatedly reading the data. Second, using line-by-line analysis, each sentence was read line-by-line. In each case, all sentences containing words or contents related to mental, physiological, social, and relational recovery were coded. Third, focusing on recurring themes of recovery, concepts were derived by grouping similar themes. “Theme” was found according to contents of recovery. “Concepts” were obtained by extracting and combining common themes from experiences related to recovery. Fourth, by continuous comparing, concepts, subcategories, and categories were derived. These concepts, subcategories, and categories derived from open coding were restructured according to the flow and order of time.

## 3. Results

### 3.1. Characteristics of Individuals

As a result of analysis, ages of those who experienced nature-based therapy ranged from teenagers to the elderly in their 80s. There were 77 men, 83 women, and 20 miscellaneous cases in which the gender was not identified or classified as other. These participants had various mental, physiological, and social symptoms (stress, depression, cancer, recovering patient, metabolic diseases, neurosis, addiction, rare and intractable disorder, interpersonal conflict, suicidal ideation, cognitive impairment, etc.). Among these symptoms, depression and stress coexisted the most (102 cases).

### 3.2. Place and Type of the Experience

Forests and parks near the place of residence were the most commonly experienced place (67.6%), followed by recreational forests, arboretums, close mountains, and remote mountain life. In this study, the park was a wooded forest and the arboretum was a national forest designed for forest healing. Remote mountain life meant that participants with diseases lived in deep mountains to recover. The duration of the recovery experience in the natural environment differed by disease and individual. In some cases, participants experienced a healing effect with just one visit. In others, participants experienced recovery with multiple visits over a long term.

It was difficult to accurately analyze the number and the duration of the visit to the natural environment. Data on the frequency of visits included daily visits, two to three times a week, irregular visits, and regular visits for more than 20 years. Experiences in the natural environment included walking, staying in a quiet place and resting, forest bathing, talking with trees, observing trees, hiking, observing animals and plants, and contemplation and meditation. As for the motivation for the visit, individuals with cancer or atopic dermatitis mentioned that they actively visit forests for the purpose of a cure. However, most people visited natural environments by chance or simply visited forests and parks just because they were near their homes. Once they had a positive experience, they visited the natural environment more actively. In particular, those who lived close to forests visited forests more frequently (almost daily) and continuously. Results indicate that frequent trips to parks or forests near one’s home are more effective for health promotion than occasional trips to distant mountains.

### 3.3. Derivation of Concepts and Categories

Through open coding, 82 concepts, 21 subcategories, and six categories were derived (Table 2).

### 3.4. Nature-Based Therapy Process

**Figure 1:**
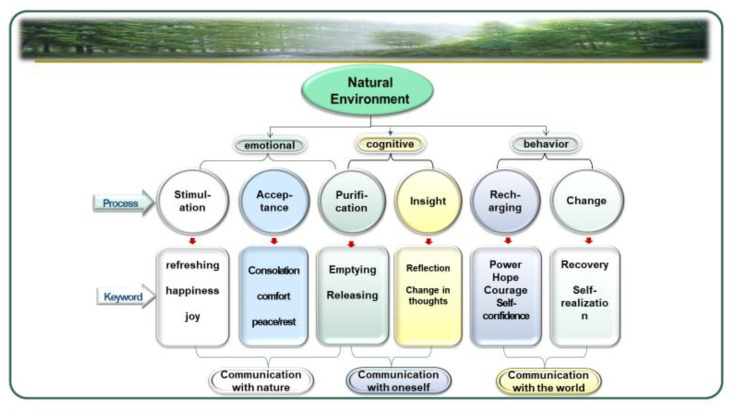
Six-Step Model of Nature-Based Therapy Process.

The nature-based therapy process is a systematic connection of healing phenomena in the natural environment. In this study, the nature-based therapy process was developed by analyzing the progress of healing according to time among the content described by people who experienced nature-based therapy. It was found that the nature-based therapy process was composed of six categories: Stimulation, acceptance, purification, insight, recharging, and change. Based on these results, a comprehensive six-step model of the nature-based therapy process was developed (Figure 1).

#### 3.4.1. Stimulation

On visiting the forest, participants felt better and refreshed by five sensory effects of the forest. They also experienced emotions such as happiness, fascination, curiosity, and joy. Their senses and sensibilities were recovered. Participants who had experienced such positive stimulation tended to visit natural environments more actively and frequently. Therefore, the experience of positive stimulation in the natural environment is an important starting point for healing and change. In the stimulus stage, words related to positive emotions such as mood, beauty, refreshment, pleasure, joy, and fascination appeared.

“Seeing the green trees, listening to the sounds of all kinds of birds and insects in my ears, and falling into the imagination as if I was in heaven when faced with the cool wind blowing from the forest.”

(2015, alcoholism)

“I climbed the mountain and walked through the forest, and I liked the sounds of the wind blowing. In particular, the sounds of the leaves striking and the sunlight shining between the green forest felt so fresh and beautiful.”

(2015, depression and stress)

“First, my ears were cleared by the birdsong. The bird sang deep enough to penetrate my lungs through my ears and stimulate my mood. ”

(2015, depression and stress)

“When I went to the forest, various fragrances, birdsongs, trees, and small pretty grass made me happy. Fresh air enters my whole body and clears my mind ... At home, it was difficult to breathe because of cancer cells, but in the forests, it was like magic because I could breathe comfortably like using an oxygen respirator.”

(2015, cancer patient)

#### 3.4.2. Acceptance

In this stage, participants experienced receptive feelings in the forest, including the sense of consolation and comportment. The forest is a place where participants could rest and relax at any time. Participants felt that the forest accepted everything about them. They felt consolation and comfort in the forest as if they were in their mother’s arms. Their tiring and exhausting lives were relieved when they communicated emotionally with the nature. Their minds were opened. In the acceptance stage, words such as friends, mother, comfort, relax, and hug gave emotional stability and consolation appeared.

“As I walked along the forest, I felt as if I was at a party invited to happiness, and I felt precious and special. ”

(2015, alcoholism)

“Walking through the forest, I feel like I’m in my mother’s arms. The forest comforts the tired and exhausted me and accepts my everything.”

(2014, depression and stress)

“I told the forest of my troubles, greed, and many heartfelt stories. The forest listened to me silently. The forest was my mother, my friend, my mentor, and myself.”

(2014, stress)

“The oxygen and phytoncide of the forest became my friend and touched my tired and painful body and mind ... Sometimes like a friend, sometimes as a mother who accepts everything, it comforted me, saying, ‘okay, okay’.”

(2015, patient)

“Holding a pine tree, which was my friend, seemed to make me lean and listen to my heart. The forest seemed to comfort me every time I visited.”

(2015, cancer patient)

#### 3.4.3. Purification

In the purification stage, participants overcame and dissolved their negative feelings. They vented and released their negative energy in a quiet forest. Their minds and emotions were then lightened and cleansed. This led them to experience relief from stress. Their pain and anger then disappeared. They also forget worries while they walked through the forest. They could honestly recognize their own feelings of avoidance in the tranquil forest alone. They confided stories from their heart that they usually could not tell others. They communicated with nature, emptying and washing away their mind and emotions. They became relaxed and generous so they could afford to look back and reflect on themselves. Purification is an important mediator that can lead to insight. Words used frequently at this stage were release, dissolve, tear, disappear, and forget.

“My mind was generous when I came down from the mountain and everything was forgiven”

(2015, alcoholism)

“When I climbed a steep mountain road … all my worries disappeared and I felt refreshed. When I climbed the mountain, my thoughts were cleared up and my head felt lighter.”

(2015, depression and stress)

“I have told the forest my worries, greed, and inner feelings … I drop the manager’s title and the dream of promotion; I started emptying everything one-by-one ... and after a year and a half, I started to feel comfortable.”

(2014, stress)

“As I visited the forest every day, my mind began to empty. Greed, hatred, and resentment in my heart disappeared ... As I walked through the forest, my resentment for the doctor disappeared and was forgiven.”

(2015, cancer patient)

“Drinking the clear night air in the forest, my body was feeling completely clean. Surprisingly, it seemed that the disease of heart that weighed me heavily was disappearing in the forest. The forest fairies seemed to blow the stress away. When I was in the forest, I forgot my anxiety, my worries disappeared, and my stress disappeared.”

(2015, mental illness)

#### 3.4.4. Insight

Insight is the most important and meaningful stage in the nature-based therapy process. Participants experienced awakening through self-reflection and meditation in nature. They then communicated with themselves and talked with their inner self. They knew what they really wanted. They found a new way of life. They regained their identity. They also discovered the meaning and purpose of their life and reinterpreted the meaning of their pain. The most important phenomenon at this stage was “change of thought”. “Change of thought” is a core phenomenon of nature-based therapy. Participants could make a choice for new life through “change of thought.” Factors that could promote insight in nature are survival methods of animals and plants, strong vitality, and the order of nature. Frequently used words at this stage were thinking, reflection, enlightening, finding a dream, and understanding.

“It was alcohol that destroyed my family, and it was alcohol that was dropped me in depression and despair. I started to think about how to live well. If I had to give up and go as I expected, my remaining life was so pitiful and unfair.”

(2015, alcoholism)

“While walking through the forest, I could talk to myself … I thought my life was the way I had to climb up myself ... Rather than waiting for someone to change me, I thought I had to change myself. While those thoughts filled my mind, I wanted to study. It’s still a difficult family environment, but in the end I felt that I had to change. I wanted to achieve the results myself.”

(2015, depression and stress)

“Communicating with the forest, I realized that all my conflicts and worries came from vain greed ... The forest was a mentor to me.”

(2014, stress)

“I enjoyed thinking naturally as I walked through the forest. As a result, I began to realize precious things. It was my family and the dream of my youth.”

(2015, stress)

“The old trees, the stones worn out over time, the grass that knows when they were bloomed became my teacher ... I realized that the mind should be healed first, not the body. I gained wisdom from many of the teachers in the forest.”

(2015, cancer patient)

#### 3.4.5. Recharging

The fifth stage is recharging. It fills participants with positive energy such as hope, courage, and confidence. Recharging involves both psychological and physiological aspects. In the natural environment, participants developed the will and desire for life. Hope, courage, confidence, and positive thoughts became energy that could overcome difficulty and create a new life. Recharged with positive energy, participants could go back into the world they were once afraid of and avoided. Words often used at this stage were power, hope, energy, courage confidence, positive, vigor, and vitality.

“Before that, I struggled with guilt for my family, I was filled with the courage and willingness to start again”

(2015, alcoholism)

“The time to stay in the mountains has increased. As I grew in strength, I grew a confident and gradually began to think positively. The mountain gave me a good energy … I certainly felt as I was going up the mountain and my lack was filling up and my mind was refreshing … The good feelings and positive energy I felt as I climbed the mountain changed me.”

(2015, depression and stress)

“As I started to go to the mountains, many negative thoughts disappeared and positive thoughts were filled. I felt good thoughts springing up like fountains.”

(2014, stress)

“I didn’t want to do anything and it was painful ... As this thought diminished, I began to desire to do something ... When I come to the mountain, my desire for life springs up.”

(2015, cancer patient)

#### 3.4.6. Change

The last stage is change. In this stage, participants recovered and changes occurred mentally and physically. Participants could now live a life with changes such as relationship restoration, re-employment, advancement, new challenges, and accomplishments. In addition, the value of life could be changed. They described having a more positive attitude, leading to a satisfying life. These changes ultimately led to self-realization. They described this life as rebirth, reproduction, and rejuvenation. Words that appeared frequently at this stage were improvement, happiness, health, healing, treatment, recovery, love, lightening, and positive.

“Since then, I have not drunken alcohol at all. Of course, there are days when I want to drink alcohol depending on my mood, but I did not even go to the supermarket to escape the temptation. For me, the best prescription was to go to a mountain where cool oxygen is emitted.”

(2015, alcoholism)

“And time went by and I took a second SAT. I just want to achieve what I want to do. There was only one thing and I passed one of the colleges I wanted. At the end of the challenge I started to get out of the dark tunnel, I achieved what I wanted to do. My personality has become much more cheerful since then, and I have the courage and mental power to boldly challenge and overcome hard work. And I’m walking hard on my way to my dream. It was unimaginable before.”

(2015, depression and stress)

“For me, forests have given me psychological stability, peace of mind, and health that I can’t get anywhere else in the world. Forests gave me a great life affordability and happiness that I can’t get or feel in gold.”

(2014, stress)

“My depression and popular avoidance were cured by 90%. Even without the psychiatric treatment ... 3–4 days in the mountains are so happy that the rest of the day has no time to depress.”

(2014, depression)

“Now I am happy every day. The ordinary daily life is special, and every day is coming up anew.”

(2014, stress)

“I became more humble. I am generous with people and things and feel grateful for the little things.”

(2014, depression and stress)

## 4. Discussion

### 4.1. Emotional, Cognitive, and Behavioral Change Process

This study found that nature-based therapy process had six categories: Stimulation, acceptance, purification, insight, recharging, and change. These six steps of nature-based therapy could be categorized into three aspects: Emotional change, cognitive change, and behavioral change (Table 3).

In the psycho-evolution theory (PET), Ulrich et al. [8] have emphasized that the natural recovery effect is associated with a shift to a more positive emotional state. Kaplan & Kaplan [9] have recognized four stages (clearing thoughts from the mind, regaining focus and attention, reducing distractions from the mind while paying attention to thoughts in silence, and setting priorities, possibilities, actions, and goals in life including reflection on one’s life) of the natural recovery experience in the attention restoration theory (ART) and emphasized aspects of attention recovery. Although results of this study seemed to differ from these previous theories, they could be considered as essentially an integration of the two previous theories. This study confirmed that recovery in the natural environment progressed through continuous interactions of emotional and cognitive changes.

The first change that participants experienced in the natural environment was a shift from negative to positive emotions. Negative emotions such as tension, anxiety, and anger were reduced while positive emotions were increased. These findings are consistent with studies showing that positive emotion is the first to appear when humans encounter a natural environment [34]. They are also consistent with previous findings showing that when humans are in a natural environment, negative emotions will decrease while positive emotions will increase [13,17,20,27,35,36,37,38].

Next, participants’ experiences in the natural environment evolved into cognitive changes. Participants changed into having positive emotions in the natural environment. Then, they began to reflect on themselves in a more relaxed and stable state. They changed their perspectives about themselves and their problems while they were in the nature. They moved on to the next stage of planning and preparing for the future.

The reason for cognitive changes in the natural environment is related to positive emotions. Positive emotions can help individuals relax psychologically and restore resources depleted by stress [39]. Positive emotions can also promote thinking and creativity to help solve daily life problems more flexibly [40]. The cognitive changes in this study are consistent with previous studies showing that the natural environment can improve one’s ability to solve daily life problems and self-regulation by reducing fatigue and improving efficiency [22,41,42]

Sonntag-Öström [43] has reported that when his patients can relax in the forest and find peace of mind, they can reflect on their feelings and lives, leading to ambitions to change circumstances of their lives. Such result shows that ‘positive emotions’ and ‘peace of mind’ in the natural environment can bring about ‘reflection’ and ‘will for change in life’, consistent with results of the present study.

The third change that participants experienced in the natural environment was their behavioral change. Those who had experienced emotional and cognitive changes were found to be healthier in body and mind than they were before. They lived healthy and happy lives with positive attitudes and behaviors. In other words, positive emotional experiences in the natural environment promoted changes in thinking and cognitive systems. Cognitive changes then brought about changes in behavior. Most participants had stress along with various mental and physical diseases. Their emotional and cognitive changes caused their body and mind to interact with each other, resulting in recovery and leading to restorations of interpersonal relationships and social life.

### 4.2. Psychological Mechanisms and Nature-Based Therapy Factors

Psychological mechanisms that cause emotional and cognitive changes in the nature-based therapy process are as follows. Sense of beauty, wonder, and freshness in the natural environment could provide psychological and emotional stimulation, evoking positive emotions and awe. Participants were immersed in various charms of the nature. They built emotional rapport with various creatures of nature. As they experienced a sense of unity with nature, their negative emotions such as rage and anxiety disappeared. When they were deeply immersed in nature, they went one step further and had a chance to reflect on themselves. Deep communication with nature took participants deeper into themselves. At this time, they could fully focus on themselves and look into their feelings and thoughts (i.e., communicate with themselves). By communicating with themselves, participants could realize the cause of their problem and decide to change. Such experience occurred mainly through reflection in silence when they were alone in the nature.

Factors triggering emotional changes in their interactions with nature were found to result from stimulation of five main senses coming from forest, such as pleasant smell, landscape, beauty, and fresh air. Factors that could promote cognitive changes were various elements of the nature, such as the natural order, the survival and robustness of animals and plants, and plants rooting and surviving in a harsh environment. Participants reflected on themselves as they observed animals, plants, and insects that exerted their utmost in a given environment, seeing trees grow their leaves, watching flowers blooming according to the time and season, and observing grass and trees surviving the cold. In addition, they develop a new perception of the situation and themselves, away from negative perspectives of their reality.

Another important element in this reflection process was that participants could discover the meaning and purpose of their life through their observations of the nature. Many participants, unable to find meaning in their hopeless lives, had the feeling of frustration and depression, addiction, and suicide attempts. However, they found their new meanings for life in the natural environment. Participants began to accept their negative and destroyed situations and self-image through their observations of trees managing to grow in a harsh environment. They saw creatures conforming to the order of nature and accepted their own painful reality. Seeing various creatures in nature finding their own ways of survival and living, people came to accept their own values. Their self-esteem was recovered. Their helpless body and mind were recovered through their changes of perspectives even in difficult circumstances. Psychological changes led to physical changes because body, mind, and emotion acted as an one system. Improved body and mind brought hope and confidence for new life, leading to better mental and physical improvements. As a result, participants who had experienced natural recovery effects described in their essays that they were living healthier and happier than before.

At this time, the initiator and driver of such changes were oneself, indicating that their mind and body were recovered through reflection on their own. In this regard, the natural environment can be a place that provides a transcendence to overcome difficulties of reality and a place of “self-therapy” that promotes finding the cause of the problem and resolving the issue by oneself.

### 4.3. Comprehensive Analysis of the Nature-Based Therapy Process

A comprehensive finding of this study was that the nature-based therapy process proceeded with a very complex and multidimensional mechanism, in which the body, mind, and emotional cognitive behaviors interacted with the nature (Table 4). Results of this study are summarized as follows.

- The nature-based therapy process consisted of six categories.
- The six-step process proceeded through emotional, cognitive, and behavioral changes.
- The psychological mechanism for nature-based therapy consisted of “communication with nature”, “communication with oneself”, and “communication with the world”. In other words, “communication with nature” could promote participants’ changes into positive emotions while “communication with oneself” could trigger cognitive changes. Participants who had experienced emotional and cognitive changes in the natural environment changed to lead a life with more intensive and healthy “communication with the world” in terms of daily activities, social lives, and interpersonal relationships.

The results of this study are consistent with Wilson’s “biophilia” hypothesis [10]. In addition, the six-step model of the nature-based therapy process presented in this study supports Ulrich’s PET theory [8] emphasizing positive emotions in nature and Kaplan’s ART theory [9] emphasizing aspects of attention recovery. Results of this study can also be considered to be an integrated linkage of many previous studies on the theory of human recovery in nature.

## 5. Conclusions

The nature-based therapy process is composed of six stages: Stimulation stage, acceptance stage, purification stage, insight stage, recharging stage, and change stage. The nature-based therapy process does not comprise a single element or step. Instead, it is an integrated way of healing through emotional and cognitive changes. In other words, in the nature-based therapy process, self-healing progresses as the mind and body interact with various elements of the nature. Natural environments are not only healing places where physical symptoms and psychological problems are healed, but also healing places where holistic healing, including oneself and others as well as social and relational recovery, can take place. In addition, natural environment, such as a forest, can serve as a place that provides a transcendence to overcome difficulties of reality and a place of “self-therapy” that can help one find the cause of the problem and resolve the issue by oneself.

This study was significant in that it derived a theoretical model of the nature-based therapy process and mechanisms. It was also meaningful in that it explored changed lives and long-term effects of participants after experiencing natural recovery. However, it has limitations in generalization since this study is based on self-reported “forest therapy experiences”. Further research is needed to establish a more systematic theoretical model through the development of nature-based therapy programs based on results of this study and structured interviews.

## Figures and Tables

**Table 1:** Four stages of the open coding process.

Coding Stage	Analysis Process	Contents
Open coding	1st analysis	Derive overall feeling and experiences
	2nd analysis	Coding
	3rd analysis	Derive a concept
	4th analysis	Derive subcategory, category

**Table 2:** Concepts and categories.

Concept	Subcategory	Category
Feeling better Happiness Enchantment, feeling beauty Fluttering, delight, fascination Joy and pleasure Feeling moved, impressed Awe, mystery, novelty	Positive emotions	Stimulation
Cool and refreshing mood Stress relief Headache disappears and clear head Fatigue disappears Feeling of clear body and mind Deep sleep Feeling peace Physical changes	Change of mind and body	
Curiosity, interest, and attention begin to develop Feeling alive Emotion has been rich and survives Feeling freedom and liberation	Recovery of emotions and senses	
Unconditional acceptance and welcome Embrace and hug Feeling of peace of mind and comfort	Peace of mind	Acceptance
Healing of wounds Consolation and serenity Become a friend and companion Just listen silently and encourage Open mind	Consolation and empathy	
Releasing anger Reveal innermost feelings Have honest conversation Tears come out	Eject suppressed feelings and emotions	Purification
Emptying and washing mind Hate and resentment disappear Releasing greed Less worry and concern Generous heart Distraction disappears Lightened mind	Relieve negative emotions	
Deep reflection and meditation Concentration occurs	Reflection and meditation	Insight
Looking back on one’s life Reflection occurs Enlightenment as to the cause and solution of the problem	Self-awareness and reflection	
Realizing life’s wisdom and reason from the providence of nature Learn lessons from the survival strategies for plants and animals	Realizing the wisdom of life	
Search for one’s inner self True meeting with oneself Have an inner conversation with oneself, including dreams Know one’s preciousness Restoration of self-confidence and self-esteem Finding real self	Identity recovery	
Recognize and accept reality and limitations Accept pain as part of life	Acceptance of reality	
Finding the meaning of life Finding meaning in pain New interpretations of life	Discover and reinterpret the meaning of life	
Increase love and understanding of others Recognize and accept children as independent beings Have a forgiving heart	Promotion and growth of self-awareness	
Control over life Rise from the past Willingness to change and choice for new life Determined to overcome difficulties	Willingness to change and choice	
Have good ideas and inspiration	Creative thinking and inspiration	Recharging
Filled with positive thoughts Filled with good energy Have strength Have confidence Increased courage and willingness to rehabilitate Hope and will of life Have motivation and vitality in life	Filled with positive energy	
Improve mental and physical health well-being life	Recovery of physical and mental health	Change
Recovery of family relationships Have a healthy social life and interpersonal relationships	Relational recovery	
Get a new job or career Challenges and accomplishments for wanted life Move to action	Active and self-directed life	
Overcome adversity and difficulties Live a thankful life To live an altruistic, shared life	Changes in attitudes and values in life	

**Table 3:** Example case of emotional, cognitive, and behavioral change processes.

Categories	Description
Emotional	“I climbed the mountain and walked through the forest, and I liked the sounds of the wind blowing. In particular, the sounds of the leaves striking and the sunlight shining between the green forest felt so fresh and beautiful ... My worries disappeared and I felt refreshed. The feeling was so good.”
Cognitive	“When I climbed the mountain, my thoughts were cleared up and my head felt lighter... I gradually got positive thoughts ... In the mountains I could talk with myself alone ... I thought I should change myself rather than waiting for someone to change me ... As those thoughts filled my mind, I wanted to study ... It’s still a difficult family environment, but I thought that I should change. I wanted to get results through hard work ...”
Behavior	“The good feelings and positive energy I felt as I climbed the mountain changed me ... And time went by and I took a second SAT.... I passed one of the colleges I wanted. It was so lonely and hard days, but I did not give up and overcome it. At the end of the challenge I started to get out of the dark tunnel, I achieved what I wanted to do. My personality has become much more cheerful since then, and I have the courage and mental power to boldly challenge and overcome hard work. And now I’m walking hard on my way to my dream. It was unimaginable before.”

**Table 4:** Comprehensive analysis of nature-based therapy mechanisms.

Phase	Phenomenon	Interaction	Mechanism	Psychological Physiological Changes
Stimulation	Positive stimulation survives feelings and emotions	Emotional change	Communication with nature	Recovery through mind and body interaction
Acceptance	Experience receptive feelings from the forest Open mind/comfort/stable			
Purification	Eruption and resolution of negative emotion			
Insight	Awareness and reflection	Cognitive change	Communication with oneself	
Recharging	Filled with positive energy			
Changing	Healing and recovery Healthy and happy life	Behavioral change	Communication with the world	
